# The lncRNA CADM2-AS1 promotes gastric cancer metastasis by binding with miR-5047 and activating NOTCH4 translation

**DOI:** 10.3389/fphar.2024.1439497

**Published:** 2024-09-06

**Authors:** Yu-Tong Zhang, Li-Juan Zhao, Teng Zhou, Jin-Yuan Zhao, Yin-Ping Geng, Qiu-Rong Zhang, Pei-Chun Sun, Wen-Chao Chen

**Affiliations:** ^1^ Department of Gastrointestinal Surgery, Henan Provincial People’s Hospital, Henan University People’s Hospital, Zhengzhou University People’s Hospital, Academy of Medical Sciences, Tianjian Laboratory of Advanced Biomedical Sciences, Zhengzhou University, Zhengzhou, China; ^2^ State Key Laboratory of Esophageal Cancer Prevention and Treatment, Key Laboratory of Advanced Drug Preparation Technologies, Ministry of Education of China, Institute of Pharmaceutical Sciences, Zhengzhou University, Zhengzhou, China; ^3^ State Key Laboratory of Esophageal Cancer Prevention and Treatment, Academy of Medical Sciences, Tianjian Laboratory of Advanced Biomedical Sciences, Zhengzhou University, Zhengzhou, China

**Keywords:** lncRNA CADM2-AS1, gastric cancer, metastasis, NOTCH4, miR-5047

## Abstract

**Background:**

Multi-organ metastasis has been the main cause of death in patients with Gastric cancer (GC). The prognosis for patients with metastasized GC is still very poor. Long noncoding RNAs (lncRNAs) always been reported to be closely related to cancer metastasis.

**Methods:**

In this paper, the aberrantly expressed lncRNA CADM2-AS1 was identified by lncRNA-sequencing in clinical lymph node metastatic GC tissues. Besides, the role of lncRNA CADM2-AS1 in cancer metastasis was detected by Transwell, Wound healing, Western Blot or other assays *in vitro* and *in vivo*. Further mechanism study was performed by RNA FISH, Dual-luciferase reporter assay and RT-qPCR. Finally, the relationship among lncRNA CADM2-AS1, miR-5047 and NOTCH4 in patient tissues was detected by RT-qPCR.

**Results:**

In this paper, the aberrantly expressed lncRNA CADM2-AS1 was identified by lncRNA-sequencing in clinical lymph node metastatic GC tissues. Besides, the role of lncRNA CADM2-AS1 in cancer metastasis was detected *in vitro* and *in vivo*. The results shown that overexpression of the lncRNA CADM2-AS1 promoted GC metastasis, while knockdown inhibited it. Further mechanism study proved that lncRNA CADM2-AS1 could sponge and silence miR-5047, which targeting mRNA was NOTCH4. Elevated expression of lncRNA CADM2-AS1 facilitate GC metastasis by up-regulating NOTCH4 mRNA level consequently. What’s more, the relationship among lncRNA CADM2-AS1, miR-5047 and NOTCH4 was further detected and verified in metastatic GC patient tissues.

**Conclusions:**

LncRNA CADM2-AS1 promoted metastasis in GC by targeting the miR-5047/NOTCH4 signaling axis, which may be a potential target for GC metastasis.

## 1 Introduction

Gastric cancer (GC) was a malignant tumor originating from the epithelial cells of the gastric mucosa (Machlowska et al., 2020). In 2022, GC has emerged as the fifth leading cause of cancer-related mortality globally, posing a grave threat to the nation’s public health (Siegel et al., 2024). According to 2020 statistics, China accounted for 44% and 48.6% of global GC cases and fatalities respectively, with the extremely high disease burden (He et al., 2024). The high incidence of local recurrence and distant metastasis was the main reason of the high mortality (Li G. Z. et al., 2022). In China, more than 80% of GC patients were locally advanced or advanced GC. The GC could metastasize not only to distant lymph nodes, liver and peritoneum, but also to lung, adrenal gland, brain and other sites (Oki et al., 2016; Ma et al., 2022; Fang et al., 2022; Kroese et al., 2022).The treatment of GC was still based on traditional methods such as surgical resection and chemotherapy, but these treatments were more suitable for patients with early GC without metastasis. The treatment of GC patients with metastasis was limited, and the prognosis was poor (Smyth et al., 2020; Thrumurthy et al., 2015). Therefore, it was essential to deeply study the development mechanism of GC metastasis, excavate the related biomarkers of GC metastasis, and propose new treatment plans for GC.

About 70% of the human genome could be transcribed, but only less than 2% of transcript could encode proteins, while almost 98% of transcript was non-coding RNA (Xue and He, 2014). Long noncoding RNAs (lncRNAs) were noncoding RNAs longer than 200 nucleotides (Xing C. et al., 2021). The present study indicated that lncRNAs played a key role in multiple biological processes such as cancer proliferation, metastasis and immunity (Li Y. et al., 2022; Li B. et al., 2022; Gao et al., 2022). Changes in the expression of lncRNAs could lead to abnormal expression of transcriptome genes, thus promoting the formation and development of cancer. For example, lncRNA H19 was not only highly expressed in colorectal cancer, breast cancer and other tumor tissues, but also significantly abnormal expression in prostate cancer and oral cancer. Moreover, the high expression of H19 shortened the survival time of cancer patients and was closely associated with poor prognosis of cancer patients (Zhang et al., 2020; Wang J. et al., 2019; Singh et al., 2021; Yang et al., 2021). The expression level of lncRNA XIST was downregulated in breast cancer, and the deletion of lncRNA XIST promoted breast cancer cells metastasize to brain (Xing F. et al., 2021). LncRNAs may have the potential to be biomarkers of cancer initiation and progression and therapeutic targets for cancer. LncRNAs could regulate the biological behavior of cancer cells by interacting with proteins, RNA or DNA. The classical pathway of lncRNAs was working as competing endogenous RNAs (ceRNAs) of miRNAs, blocking the inhibitory effect of miRNAs on mRNA by reducing the binding of microRNAs (miRNAs) to 3′UTR regions of target genes mRNAs. At the same time, as a miRNA sponge, it also reduced the expression of miRNAs (Wang L. et al., 2019; Braga et al., 2020; Han et al., 2020). For instance, the upregulated lncRNA CCAT1 competitively bound to miR-28-5P, inhibiting its anticancer effect and promoting prostate cancer cell proliferation (You et al., 2019). LncRNA MIAT competitively bound to miR-50-5p, inhibiting the function of miR-50-5p to downregulate EZH2 protein, and promoted the invasion and metastasis of papillary thyroid carcinoma (Guo et al., 2021). LncRNAs interferes target genes mainly through miRNAs, following play their biological functions to participate in the occurrence and development of tumors.

There were a large number of lncRNAs, but the current functional studies of lncRNAs in GC often focused on a small number of classical lncRNAs (Sun et al., 2021; Xu et al., 2021; Syllaios et al., 2021). However, most of these lncRNAs have not yet been well studied and applicated in clinic. It’s imperative to develop some potential and deeply studied lncRNAs for clinical application. Therefore, we screen the abnormally expressed lncRNAs in metastatic GC based on clinical samples and analysis in database. LncRNA CADM2-AS1 was found to be abnormally expressed in lymph node metastatic GC tissues. Thus, we focus on lncRNA CADM2-AS1 and further study its role and mechanism in GC metastasis in this paper. By combining with clinical samples, it was expected to identify new potential lncRNAs involved in the metastasis process of GC, providing new ideas for the clinical application of lncRNAs in GC metastasis.

## 2 Materials and methods

### 2.1 Cells

GC cell line was purchased from the Cell Bank of the Shanghai Institute of Cell Biology, Chinese Academy of Sciences. The cells were maintained in Roswell Park Memorial Institute (RPMI) 1,640 medium (BI, Israel), supplemented with 10% fetal bovine serum (BI, Israel). All cells were cultured in a humidified atmosphere at 5% CO_2_ and 37°C.

### 2.2 Transfection assays

The antisense oligonucleotide (ASO) to knock down lncRNA CADM2-AS1 expression was produced and packaged by Guangzhou RIBOBIO Biotechnology Co. LTD., China; the siRNA to knock down NOTCH4 mRNA was produced and packaged by Suzhou Genepharma Co. LTD., China. The ASO/siRNA transfection was performed as follows, the HGC-27/BGC-823 cells (5 × 10^5^ cells/well) were seeded in 6‐well plates and cultured overnight in RPMI‐1640 medium supplemented with 10% FBS. The ASO/siRNA was added to 1 mL blank medium at a final concentration of 100 nM. After 12 h of co-incubation with cells, the blank medium was replaced with complete medium, and the incubation continued for 24–48 h.

### 2.3 Infection assays

The lentiviral vector containing lncRNA CADM2-AS1/miR-5047 to overexpress was produced and packaged by Shanghai Genechem Co. Ltd., China. The lentivirus infection was performed as follows, the HGC-27/BGC-823 cells (4 × 10^3^ cells/well) were seeded in 96‐well plates and cultured with RPMI‐1640 medium containing 10% FBS. The lentivirus was added to 100 μL complete medium at a final concentration of 10^7^ TU/mL. After 20 h of incubation, the lentivirus‐containing medium was replaced with complete medium. After 2 days 2 infection, the cells were incubated with 0.5 μg/mL puromycin in culture medium for 2 days to select the stable pCADM2-AS1/pmiR-5047 cell line.

### 2.4 Quantitative reverse transcription PCR (RT-qPCR)

The total mRNA was extracted by Total RNA Isolation Kit (Beibei Biotechnology, China). Then, each mRNA sample was reverse transcribed into cDNAs using lnRcute lncRNA First-Strand cDNA Kit (TIANGEN Biotechnology, China), HiScript II Q RT SuperMix for qPCR (Vazyme, China), riboSCRIPT Reverse Transcription Kit (RIBOBIO Biotechnology, China). The relative RNA levels of lncRNA CADM2-AS1, NOTCH4, has-miR-5047, has-miR-5706, has-miR-1184, and has-miR-1301-3p were detected with indicated primers. The primers of lncRNA CADM2-AS1, has-miR-5047, has-miR-5706, has-miR-1184, and has-miR-1301-3p were designed by RIBOBIO Biotechnology. The primer of NOTCH4 was designed by Tsingke Biotechnology.

### 2.5 Transwell assays

For silencing of lncRNA CADM2-AS1 (NC or ASO-CADM2-AS1) and NOTCH4 (NC or siNOTCH4), 8 × 10^3^ HGC-27 or BGC-823 cells were seeded in the upper transwell chambers. For overexpression of lncRNA CADM2-AS1 (CON or pCADM2-AS1) and miR-5047 (CON or pmiR-5047), 8 × 10^3^ HGC-27 or BGC-823 cells were added in the chambers. The medium containing 10% FBS (700 μL) was added to the lower wells. After 48 h, the cells in upper chambers were removed by cotton swab, and they migrated to the lower wells through pores were fixed with 4% paraformaldehyde and stained with 4,6-diamidino-2-phenylindole (DAPI) or 0.2% crystal violet solution, which followed by observing and counting with microscope.

### 2.6 Wound healing assay

For Wound healing assay, 5 × 10^5^ cells were seeded in 6-well. A wound was made in each well 24 h later. Plates were imaged every 24 h. Wound width was imaged and measured bymicroscope (Nikon, Japan).

### 2.7 Western blot

The cell total protein was extracted with Native lysis Buffer (Solarbio, China), adding PSMF and protein enzyme inhibitors (Meilunbio, China), which followed by degeneration with 6 × Loading buffer for 10 min at 100°C. Western Blot was performed according to the standard methods. Approximately 30 μg of protein was subjected to sodium dodecyl sulfate‐polyacrylamide gel electrophoresis (SDS–PAGE) gel. The resolved proteins were transferred to a 0.2‐μm nitrocellulose membrane (Pall, United States). The membrane carrying proteins was blocked with 5% milk in PBS for 2 h, following by first-antibody incubation like anti‐CDH1 (CST, United States), anti‐N-cadherin (CST, United States), anti‐snail (CST, United States), anti‐vimentin (Abcam, England), and anti‐GAPDH (Hangzhou Goodhere Biotechnology, China), overnight at 4°C. Next, the membrane was washed with PBS containing 0.05% Tween‐20 (PBST) at room temperature and incubated with peroxidase‐conjugated goat anti‐rabbit IgG (ZEN-BIOSCIENCE, China) for 2 h at room temperature. The membrane was then washed with PBST at room temperature and developed using an enhanced chemiluminescence reagent (Meilunbio, China).

### 2.8 MTT assay

Cell proliferation was detected by the 3-(4,5-Dimethyl-2-Thiazolyl)-2,5-Diphenyl Tetrazolium Bromide (MTT, Meilunbio, China) method. The cells were seeded and grown in 96-well plates. After the grown period, 20 μL MTT solution was added to each well and incubated with cells for 4 h. Then the supernatant was discarded and 200 μL Dimethyl sulfoxide (DMSO, Meilunbio, China) was added to each well. The absorbance was measured with microplate reader (Envision, PerkinElmer, United States) at 570 nm.

### 2.9 RNA FISH

HGC-27 or BGC-823 cells were seeded on cell climbing piece and cultured overnight in 24-well plates. Then the cells were fixed in 4% paraformaldehyde for 15–20 min. The location of lncRNA CADM2-AS1 was detected by RNA FISH SA-Biotin amplification System Kit, which was purchased from Genepharma (Suzhou, China). The general operation was as follows: Cells were permeabilized with 0.1% Buffer A, which followed by blocking with 1 × blocking solution for 30 min. The blocked cells then incubated with appropriate probe overnight. After the incubation, cells were washed with 2 × Buffer C for three times, and nuclei were stained with DAPI. Finally, the cells were imaged using a Nikon C2 Plus confocal microscope (Nikon, Japan), lncRNA CADM2-AS1, Red; miR-5047, Green; nuclei, Blue.

### 2.10 Dual-luciferase reporter assay

The binding sites of NOTCH4 mRNA and miR-5047 were predicted by the Targetscan database (https://www.targetscan.org/vert_80/) (Mcgeary et al., 2019). The binding sites of lncRNA CADM2-AS1 and miR-5047 were predicted by the RNA22 database (https://cm.jefferson.edu/rna22/Interactive/RNA22Controller) (Loher and RIGOUTSOS, 2012). The lncRNA CADM2-AS1 and NOTCH4 mRNA serial truncations were cloned into pmirGLO vector, which were purchased from Suzhou Genepharma Co. LTD. Then, 2 μg reporter vector, 2 μg ectopic expression vector and 2 μg pmirGLO vector were transfected into 293T cells with EntransterTM-H4000 (Engreen, China). For miR-5047 stability assay, 2 μg reporter vector and 100 nmol mimic (Genepharma, China) were co-transfected into 293T cells with EntransterTM-H4000. About 48 h later, cells were lysed in 500 μL of passive lysis buffer. The firefly luciferase activity and the Renilla activity were determined by Dual-Luciferase^®^ Reporter Assay System (Promega, United States). For each experiment, the firefly luciferase activity was normalized by Renilla activity.

### 2.11 *In vivo* assay

Five-week-old male NOD/SCID immunodeficiency mice were purchased from the Gempharmatech Co., Ltd., China. All animals were housed in a pathogen-free environment, and the experimental protocols were approved by the Ethics Committee of Zhengzhou University Health Science Center. The mice were randomized into three groups (n = 7 in each group). 5 × 10^5^ or 7 × 10^6^ HGC-27 cells were diluted in 200 μL of sterile PBS and injected through tail vein (5 × 10^5^ for the animal experiment in Figure 3, and 5 × 10^6^ for the animal experiment in Figure 7), and the mice were weighted every week. After 8 weeks, mice were euthanized and dissected to detect the GC cell metastasis *in vivo*.

### 2.12 Hematoxylin-eosin staining (HE staining)

The lung tissues of the mice were dissected and fixed in 10% buffered formalin solution, which followed by embedding in paraffin wax. The serial sections (5 μm) were cut from the tissue blocks, deparaffinized in xylene, and hydrated in an alcohol series (75%, 85%, 95%, and 100%). The tissue sections were then incubated with hematoxylin (Solarbio, China) and eosin (Solarbio, China), which staining cell nuclei and cytoplasm separately. Finally, the sections were dehydrated in an alcohol series (75%, 85%, 95%, and 100%) and xylene. The HE stained sections were imaged and analyzed with a microscope (Nikon, Japan).

### 2.13 Patients tissue specimens

GC tissues and adjacent tissues of 60 patients were collected from the Henan Provincial people’s Hospital. All tissue’s collections were authorized by the Ethics Committee of Zhengzhou University People’s Hospital (approval date 2020, code 172). Detailed characteristics of patients were showed in Supplementary Table 1.

### 2.14 Statistical analysis

Three independent trials were performed for each *in vitro* experiment. Spearman’s correlation coefficient was used to evaluate the correlation between two groups. RNA sequencing and GC tissues analysis were performed by using Volcano and BOX plot tools in Hiplot Pro (https://hiplot.com.cn/), a comprehensive web service for biomedical data analysis and visualization. All statistical analyses were performed using GraphPad 8.0. The data difference was analyzed using Student’s *t*‐test. The differences were considered significant at *p* < 0.05. *p < 0.05, **p < 0.01, ***p < 0.001, ****p < 0.0001.

## 3 Results

### 3.1 LncRNA CADM2-AS1 overexpressed in metastatic GC

**FIGURE 1:**
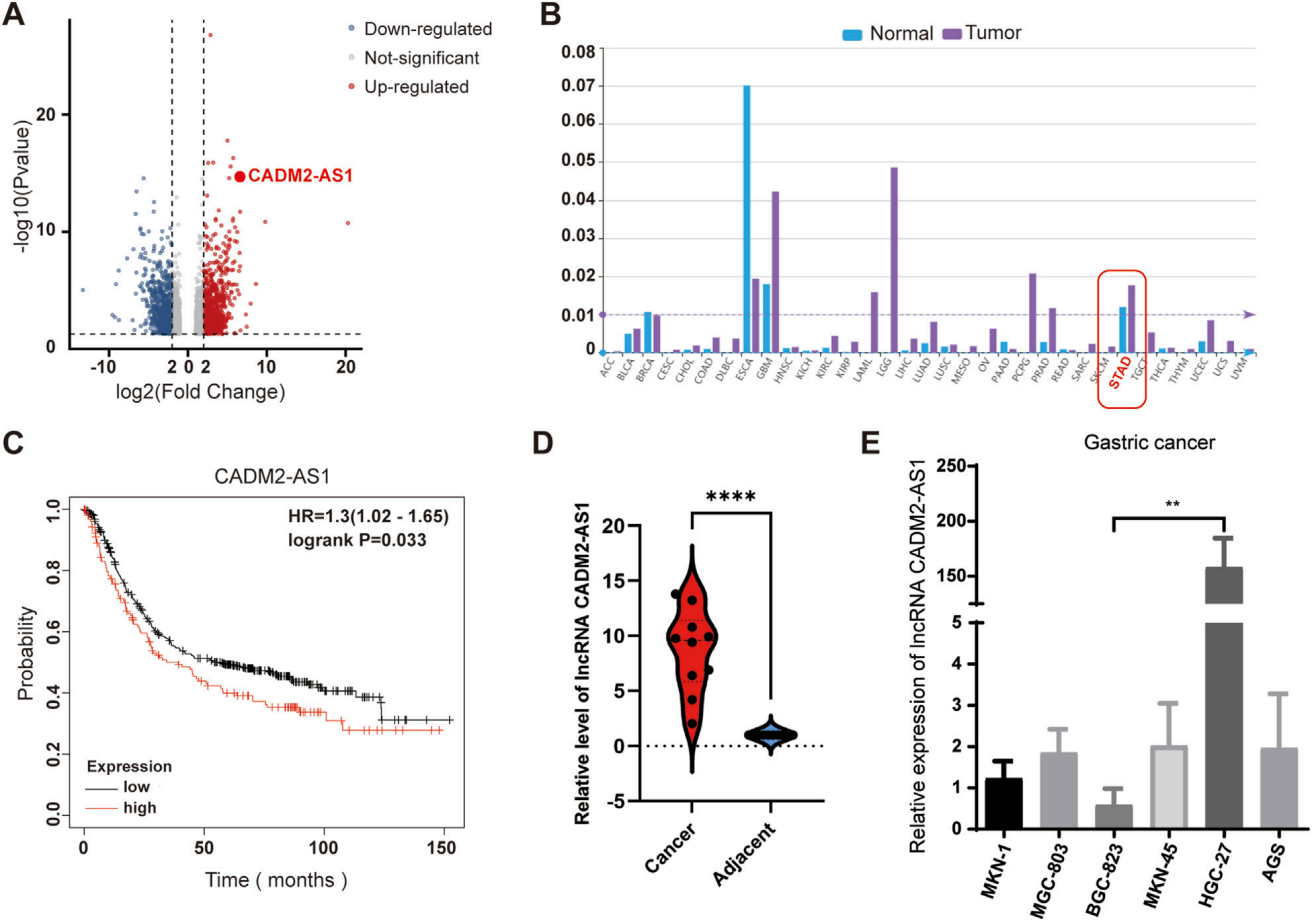
Overexpression of lncRNA CADM2-AS1 in metastatic GC. **(A)** Volcano plot of lncRNA sequencing of clinical GC tissues with lymph node metastasis and normal samples; **(B)** LncRNA CADM2-AS1 was significantly overexpressed in GC in LncRNASNP-human database; **(C)** The analysis in Kaplan Meier plotter database showed that high expression of lncRNA CADM2-AS1 was associated with poor prognosis in patients with GC. **(D)** The overexpression of lncRNA CADM2-AS1 in GC tissues with lymph node metastasis was detected and verified by RT-qPCR. **(E)** The expression of lncRNA CADM2-AS1 in GC cell lines was detected by RT-qPCR assay; n = 3, **p < 0.01.

To develop and explore lncRNAs related to the GC metastasis, GC tissues with lymph node metastasis and adjacent tissues were collected for lncRNA sequencing. The results showed that multiple lncRNAs were abnormally expressed in GC tissues, and significantly differentially expressed lncRNA CADM2-AS1 was found (Figure 1A). For a preliminary understanding of lncRNA CADM2-AS1, we start with a preliminary validation in the lncRNASNP-human database (http://bioinfo.life.hust.edu.cn/lncRNASNP/#!/), and the database displayed that lncRNA CADM2-AS1 was significantly highly expressed in GC (Figure 1B) (Miao et al., 2018; Yang et al., 2023). Besides, Kaplan Meier plotter database (http://kmplot.com/analysis/) showed that high expression of lncRNA CADM2-AS1 was associated with poor prognosis of GC patients (Figure 1C) (Győrffy, 2021). Then, the overexpression of lncRNA CADM2-AS1 in GC tissues with lymph node metastasis was detected and verified by RT-qPCR, and the result was shown in Figure 1D. It was speculated that lncRNA CADM2-AS1 may be involved in GC metastasis. To explore the differential expression of lncRNA CADM2-AS1 in GC cell lines, total RNA was extracted from GC cells (AGS, BGC-823, HGC-27, MKN-1, MKN-45 and MGC-803). The result of RT-qPCR shown that lncRNA CADM2-AS1 was highly expressed in HGC-27 and low in BGC-823 (Figure 1E). Therefore, HGC-27 and BGC-823 cells were used as cell models to explore the regulatory effects of lncRNA CADM2-AS1 on metastasis of GC.

### 3.2 Knockdown of lncRNA CADM2-AS1 inhibited the migration of GC cells

**FIGURE 2:**
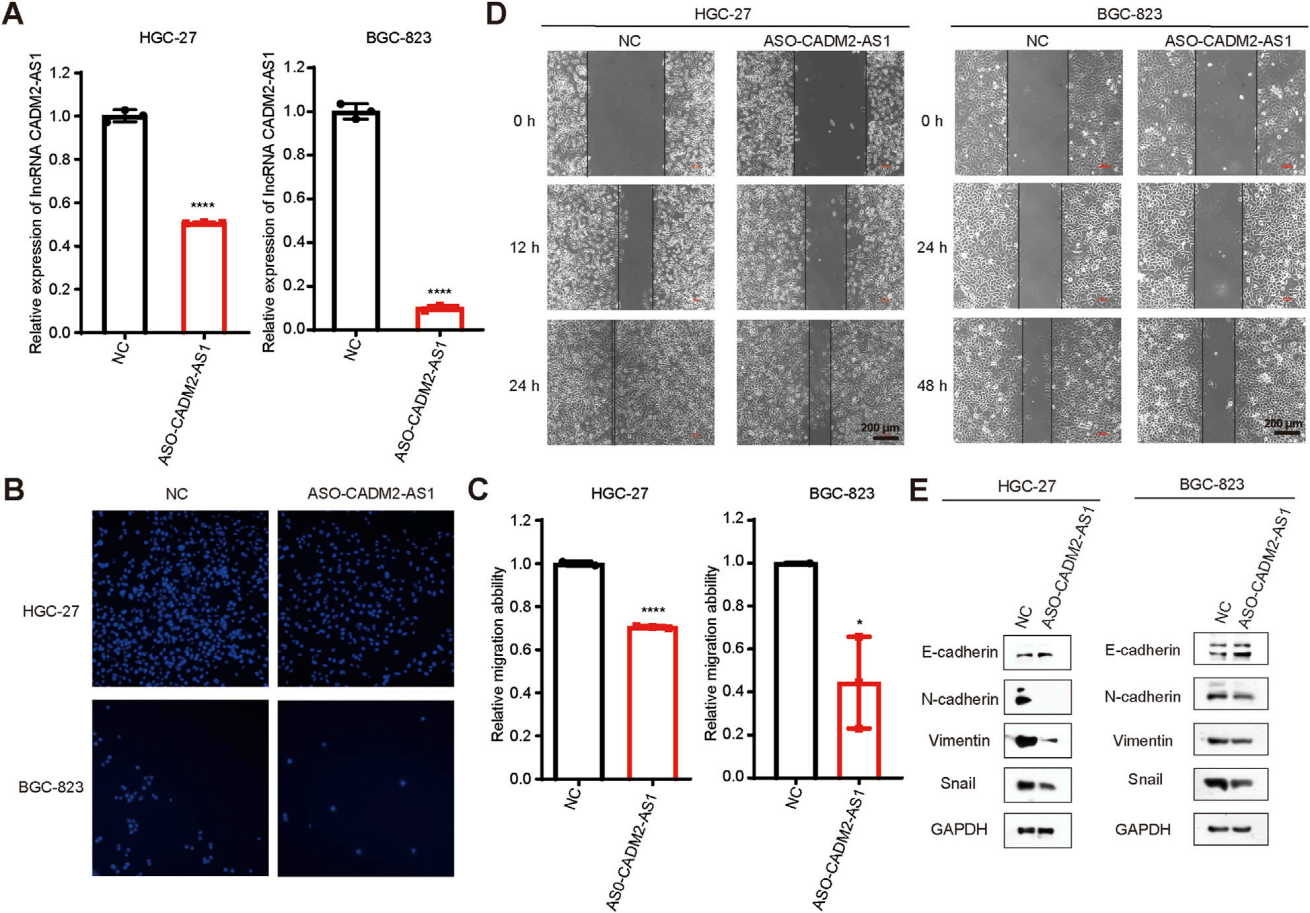
Knockdown of lncRNA CADM2-AS1 inhibited the migration activities of GC cells. **(A)** The expression of lncRNA CADM2-AS1 in HGC-27 and BGC-823 was detected by RT-qPCR assay; **(B)** Transwell verified that after knocking down lncRNA CADM2-AS1, the migration ability of HGC-27 and BGC-823 was reduced; **(C)** Figure C was the quantitative statistics of figure B; **(D)** Wound healing assay verified that knocking down lncRNA CADM2-AS1 reduced the speed of wound healing in HGC-27 and BGC-823, scale = 200 μm; **(E)** The markers of EMT progress were detected in HGC-27and BGC-823 with different conditions by Western Blot; n = 3, *p < 0.05, ****p < 0.0001, ****p < 0.001.

To investigate the effect of lncRNA CADM2-AS1 on the metastasis of GC cells, lncRNA CADM2-AS1 was knocked down in HGC-27 and BGC-823 by ASO (HGC-27 ASO-CADM2-AS1 and BGC-823 ASO-CADM2-AS1) (Figure 2A). With knocking down of lncRNA CADM2-AS1, the ability of HGC-27 and BGC-823 to invade through the Transwell membrane was reduced (Figures 2B, C). Besides, the wound healing ability of HGC-27 and BGC-823 was also diminished when lncRNA CADM2-AS1 was knocked down (Figure 2D). Meanwhile, with lncRNA CADM2-AS1 knocking down, the expression of epithelial cell marker (E-cadherin) was increased but the expression of mesenchymal cell markers (N-cadherin, Vimentin, Snail) was decreased, which were the markers of inhibited epithelial-mesenchymal transition (EMT) progress (Figure 2E). All these results shown that lncRNA CADM2-AS1 knockdown hindered the migration of GC cells.

### 3.3 Overexpression of lncRNA CADM2-AS1 promoted the migration of GC cells *in vitro* and *in vivo*

**FIGURE 3:**
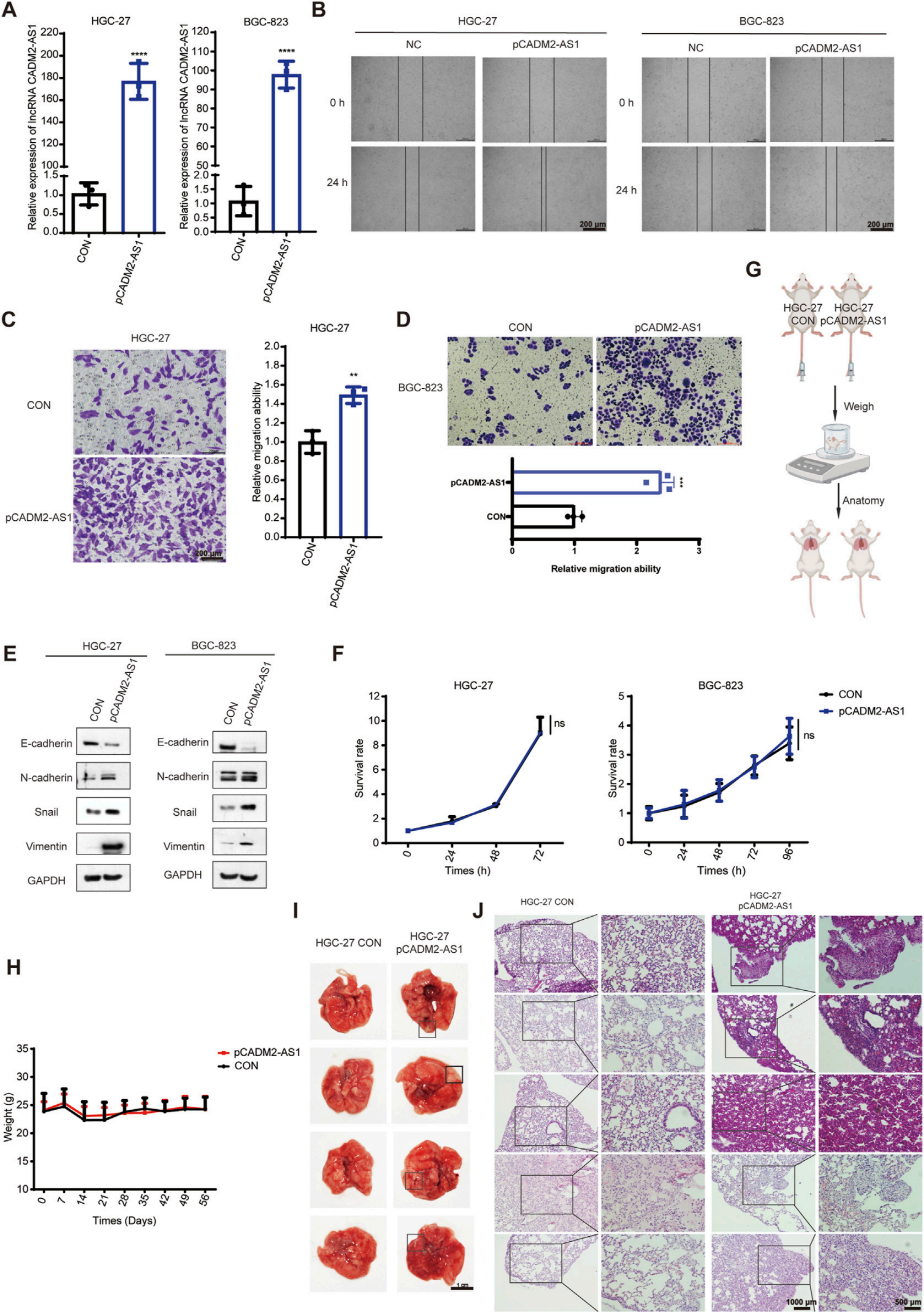
LncRNA CADM2-AS1 promoted the migration activities of GC cells. **(A)** The expression of lncRNA CADM2-AS1 in HGC-27 and BGC-823 with lentivirus infection were detected by RT-qPCR assay; **(B)** Wound healing assay was performed to detected the migration ability of HGC-27 and BGC-823 in different conditions, scale = 200 μm; **(C,D)** The effect of lncRNA CADM2-AS1 on migration ability of HGC-27 and BGC-823 was detected by Transwell assay, scale = 200 μm; **(E)** The markers of EMT progress were detected in HGC-27and BGC-823 with different conditions by Western Blot; **(F)** MTT assay was performed to detect proliferation of CON and pCADM2-AS1 cells in 96 h; n = 3; **(G)** The construction pattern diagram of mouse lung metastasis model; **(H)** The mouse in pCADM2-AS1 group and CON group was recorded; **(I)** Brightfield map of fresh lung tissues in pCADM2-AS1 group and CON group, scale = 1 cm; **(J)** Images of HE staining of lung tissues sections in pCADM2-AS1 group and CON group, scale = 1,000 μm; The right side figures were local magnification view of left side figures, scale = 500 μm; n = 6. ns, not statistically significant, **p < 0.01, ****p < 0.0001.

To confirm that lncRNA CADM2-AS1 could promote metastasis in GC cells, the lncRNA CADM2-AS1 overexpression cell models were constructed by lentiviral infection (HGC-27 pCADM2-AS1 and BGC-823 pCADM2-AS1) (Figure 3A). Compared to CON, the ability of HGC-27 and BGC-823 to invade through the Transwell membrane was increased with lncRNA CADM2-AS1 overexpression and the wound healing ability of HGC-27 and BGC-823 was also enhanced (Figures 3B–D). Similarly, the overexpression of lncRNA CADM2-AS1 downregulated the expression of epithelial cell marker (E-cadherin) and upregulated the expression of mesenchymal cell markers (N-cadherin, Vimentin, Snail) (Figure 3E). With the consideration of the cell proliferation may affect cell migration ability, we also detected the effect of lncRNA CADM2-AS1 on the proliferation of GC cells by MTT assay. The results showed that the overexpression of lncRNA CADM2-AS1 had no significant effect on cell proliferation of HGC-27 and BGC-823 cells (Figure 3F). It was further demonstrated that lncRNA CADM2-AS1 overexpression could promote the migration ability of GC cells.

To explore whether lncRNA CADM2-AS1 overexpression had the promoting metastasis biological effect of GC cells *in vivo*, we established the lung metastasis models in NOD/SCID mouse by injecting HGC-27 CON and pCADM2-AS1 through tail vein (Figure 3G). Compared to CON group, there was no conspicuous change of weight in pCADM2-AS1 group (Figure 3H). However, 8 weeks later, it was found that the lung nodules were formed in the lungs of pCADM2-AS1 group (Figure 3I). Besides, the results of HE staining shown that the nuclei were large and stained deeply, the normal structure of the lung was destroyed, and the number of pulmonary nodules were increased in pCADM2-AS1 group, which proved that the metastasis ability of GC cells was significantly enhanced in pCADM2-AS1 group (Figure 3J). All these data proved that overexpression of lncRNA CADM2-AS1 could promote the metastasis of GC cells *in vivo*.

### 3.4 Overexpression of lncRNA CADM2-AS1 promotes metastasis in GC by upregulating NOTCH4 mRNA

**FIGURE 4:**
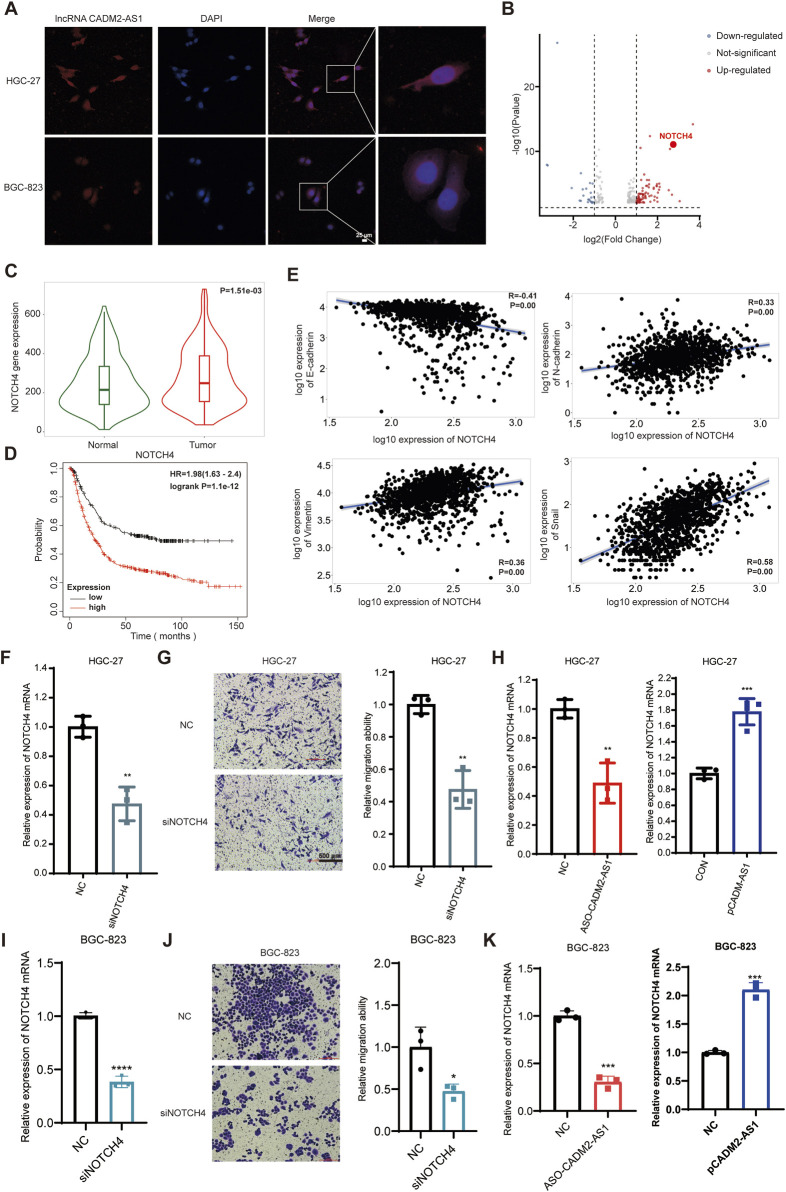
LncRNA CADM2-AS1 promote metastasis in GC by upregulating NOTCH4 mRNA. **(A)** RNA FISH assay was performed to detect subcellular localization of lncRNA CADM2-AS1 in HGC-27 and BGC-823, scale = 25 μm; Red, the Cy3 fluorescent probe represented lncRNA CADM2-AS1; Blue, DAPI represented nucleus; Merge, a merge channel graph; **(B)** Volcano plot of RNA sequencing result in HGC CON and HGC-27 pCADM2-AS1 cells; n = 3, *p* < 0.05; **(C)** The expression of NOTCH4 in GC tissues and normal tissues was searched in TNMplot database; **(D)** The relationship between NOTCH4 and prognosis in GC patients was searched in Kaplan Meier plotter database; **(E)** The relationships between NOTCH4 and EMT markers were searched in TNMplot database respectively; **(F)** RT-qPCR assay was performed to detect NOTCH4 knockdown efficiency in HGC-27 after transfecting siRNA; **(G)** The effect of NOTCH4 on migration ability of HGC-27 was detected by Transwell assay, scale = 100 μm; **(H)** The effect of lncRNA CADM2-AS1 on NOTCH4 in HGC-27 was detected by RT-qPCR assay; **(I)** RT-qPCR assay was performed to detect NOTCH4 knockdown efficiency in BGC-823 after transfecting siRNA; **(J)** The effect of NOTCH4 on migration ability of BGC-823 was detected by Transwell assay, scale = 100 μm; **(K)** The effect of lncRNA CADM2-AS1 on NOTCH4 in BGC-823 was detected by RT-qPCR assay; n = 3, **p < 0.01, ***p < 0.001.

Based on the above experiments, it has proved that the lncRNA CADM2-AS1 could promote metastasis of GC cells *in vitro* and *vivo*. However, the mechanism of lncRNA CADM2-AS1 promoting the metastasis of GC cells was still unclear, which need to be further explored. Due to different localization in cells, lncRNAs played biological functions in different ways. The result of RNA FISH assay revealed that lncRNA CADM2-AS1 was found in both the cytoplasm and nucleus of HGC-27 and BGC-823 (Figure 4A). LncRNAs competed with mRNA to bind to miRNAs, which is a classical molecular pathway for intracellular lncRNAs to function. Therefore, it is pondered that lncRNA CADM2-AS1 may encourage the metastasis of GC cells by regulating metastasis-related mRNA expression through miRNAs.

To analyze whether lncRNA CADM2-AS1 fostered metastasis of GC cells by regulating metastasis-related mRNA expression, HGC-27 pCADM2-AS1 and HGC CON GC cells were subjected to RNA-seq. It was found that mRNA levels of neurogenic locus notch homolog protein 4 (NOTCH4) was obviously upregulated after lncRNA CADM2-AS1 overexpression, which is a close regulator of tumor metastasis (Figure 4B). NOTCH4, with two intracellular and extracellular domains, is one of the four transmembrane receptors of NOTCH family (Xiu et al., 2021; Katoh and KATOH, 2020). NOTCH4 is activated binding to its corresponding ligand to participate in the NOTCH pathway, closely involving in regulating the EMT process and promoting the invasion and metastasis of colorectal cancer, breast cancer, melanoma, lung adenocarcinoma and other cancers (Zhou et al., 2020; Lin et al., 2016; Li et al., 2021; Scheurlen et al., 2022; Wu et al., 2018).

Based on the close relationship between NOTCH4 and tumor metastasis, we verify the relationship between NOTCH4 and metastasis of GC firstly. It was discovered that NOTCH4 expression in GC was higher than it in paracancerous tissues with 273 pairs of GC samples in TNMplot database (https://tnmplot.com/analysis/) (Figure 4C) (Bartha and GYŐRFFY, 2021). Moreover, high expression of NOTCH4 was associated with poor prognosis of GC patients in Kaplan Meier plotter database (Figure 4D). In addition, it was initiated NOTCH4 was negatively correlated with epithelial cell marker (E-cadherin), and was positively correlated with mesenchymal cell markers (N-cadherin, vimentin and Snail) by TNMplot database (Figure 4E). These data provide preliminary evidence that NOTCH4 contributes to the metastasis of GC. Then, we construct NOTCH4 knockdown GC cell line with NOTCH4 siRNA to verify the role of NOTCH4 in GC metastasis (Figure 4F). Compared to HGC-27 NC, the ability of HGC-27 siNOTCH4 to invade through the Transwell membrane was reduced (Figure 4G). Meanwhile, similar results were also obtained in BGC-823 cells (Figures 4I, J). All these results confirmed that NOTCH4 promoted GC metastasis. To verify the regulatory effect of lncRNA CADM2-AS1 on NOTCH4, RT-qPCR assay was performed. It was discovered that lncRNA CADM2-AS1 knockdown downregulated the level of NOTCH4 mRNA, while lncRNA CADM2-AS1 overexpression upregulated it (Figures 4H, K). These results proved that lncRNA CADM2-AS1 facilitated GC cell metastasis by raising NOTCH4 mRNA expression, but the molecular mechanism was still unknown.

### 3.5 LncRNA CADM2-AS1 upregulates NOTCH4 mRNA by decreasing miR-5047

**FIGURE 5:**
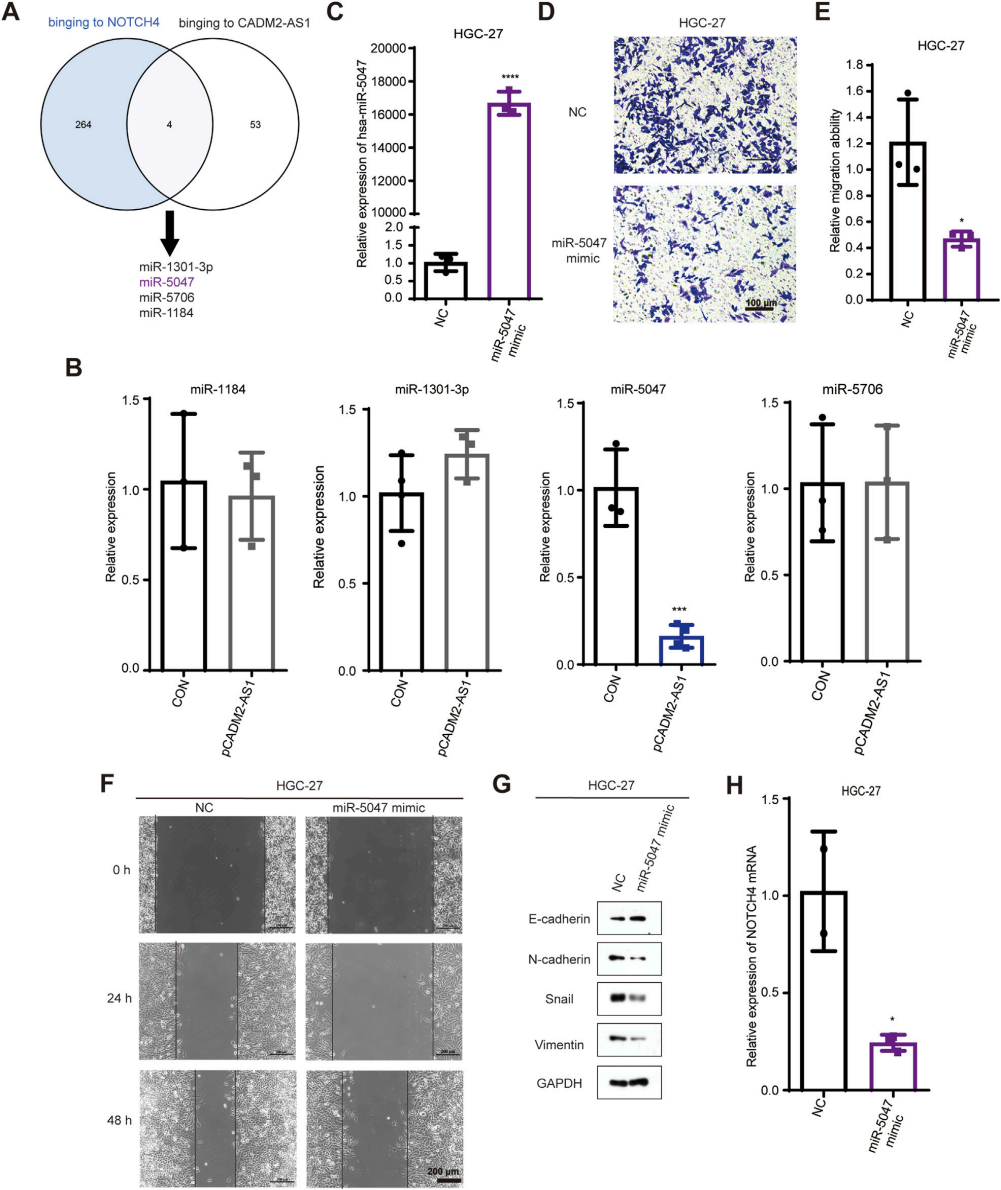
MiR-5047 may be the mediator of lncRNA CADM2-AS1 upregulation of NOTCH4 in promoting GC metastasis. **(A)** Venn Diagram of 268 miRNAs bound to NOTCH4 mRNA and 57 miRNAs bound to lncRNA CADM2-AS1 retrieved from database respectively; **(B)** RT-qPCR detected the expression of miR-1301 -3p, miR-5047, miR-5706 and miR-1184 in HGC-27 pCADM2-AS1 cells; **(C)** MiR-5047 overexpression was detected by RT-qPCR in HGC-27 which transfected with miR-5047 mimic; **(D)** The migration ability of HGC-27 with miR-5047 mimic was detected by Transwell assay, scale = 100 μm; **(E)** Figure E was the quantitative statistics of figure D; **(F)** The migration ability of HGC-27 with miR-5047 overexpression was detected by Wound healing assay, scale = 200 μm; **(G)** The expression of EMT markers in HGC-27 with the overexpression of miR-5047 were detected by Western Blot; **(H)** RT-qPCR verified the level of NOTCH4 mRNA in HGC-27 cells with overexpression of miR-5047; n = 3, *p < 0.05, ***p < 0.001, ****p < 0.0001.

To inspect the molecular mechanism of lncRNA CADM2-AS1 regulation on NOTCH4 mRNA, we search for the potential miRNAs who both bind to lncRNA CADM2-AS1 and NOTCH4 mRNA in database. There were 268 miRNAs binding to NOTCH4 mRNA were retrieved from Targetscan database (https://www.targetscan. Org/vert_80/), and 57 miRNAs binding to lncRNA CADM2-AS1 were retrieved from lncRNASNP2 database (http://bioinfo.life.hust.edu.cn/lncRNASNP/#!/); Among them, a total of 4 miRNAs (miR-1301-3p, miR-5047, miR-5706, and miR-1184) bound to lncRNA CADM2-AS1 and NOTCH4 mRNA simultaneously (Figure 5A) (Mcgeary et al., 2019; Miao et al., 2018). To verify whether lncRNA CADM2-AS1 can competitively bind to the above miRNAs, the above four miRNAs were detected by RT-qPCR with overexpression of lncRNA CADM2-AS1 firstly. Compared to CON, there were no significant differences in the level of miR-1301-3p, miR-5706 and miR-1184 when lncRNA CADM2-AS1 overexpression, while the level of miR-5047 was downregulated in HGC-27 pCADM2-AS1 (Figure 5B). The significant regulatory effect of lncRNA CADM2-AS1 on miR-5047 prompted us to speculate that miR-5047 may be the intermediate molecule between lncRNA CADM2-AS1 and NOTCH4 during the metastasis of GC.

The existing literature indicates that miR-5047 was a key negative regulator of osteosarcoma cell stemness and metastasis by target SOX2 mRNA (Chen et al., 2020). Similarly, miR-5047 significantly inhibit cervical cancer metastasis and chemoresistance by down-regulating VEGFA (Guo et al., 2019). Therefore, miR-5047 is closely related to tumor metastasis, which may also participate the metastasis of GC caused by lncRNA CADM2-AS1 overexpression.

To validate the inference of miR-5047, we detected the regulatory function of miR-5047 on the metastatic ability in GC cells firstly. The overexpression of miR-5047 GC cell line was constructed with miR-5047 mimic (Figure 5C). Besides, the result of Transwell showed that overexpression of miR-5047 inhibited the migration ability of GC cells (Figures 5D, E). The wound healing ability of HGC-27 pmiR-5047 was also weakened (Figure 5F). Meanwhile, transfecting miR-5047 mimic, the expression of epithelial cell marker (E-cadherin) was increased but the expression of mesenchymal cell markers (N-cadherin, Vimentin, Snail) was decreased when miR-5047 mimic was transfected in HGC-27 cells (Figure 5G). The overexpression of miR-5047 hindered the metastasis of GC cells. After confirming the metastasis inhibiting biological function of miR-5047 in GC, we further detected the regulatory effect of miR-5047 on NOTCH4 mRNA by RT-qPCR. The result shown that the expression of NOTCH4 mRNA was decreased while miR-5047 overexpression, which suggested that NOTCH4 was a potential target mRNA of miR-5047 (Figure 5H).

**FIGURE 6:**
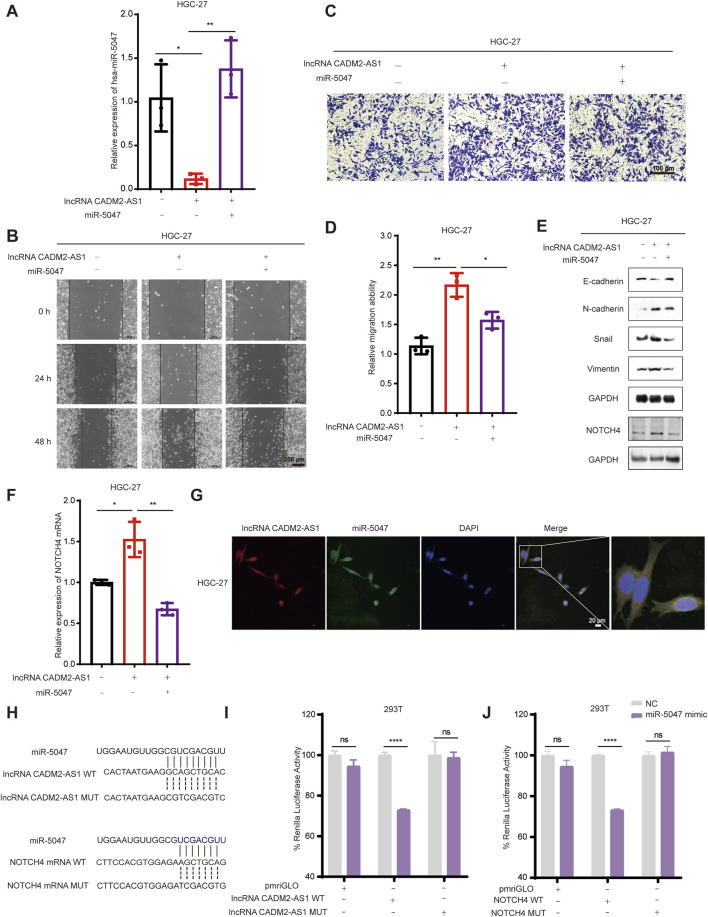
LncRNA CADM2-AS1 promoted GC metastasis by targeting the miR-5047/NOTCH4 signaling axis. **(A)** RT-qPCR assay was performed to detect miR-5047 overexpression efficiency in HGC-27 pCADM2-AS1 cell after transfecting miR-5047 mimic; **(B)** Wound healing assay was performed to detect the migration ability of HGC-27 cells in different conditions, scale = 200 μm; **(C)** Transwell assay was performed to detect the migration ability of HGC-27 cells in different conditions, scale = 100 μm; **(D)** Figure D was the quantitative statistics of figure C; **(E)** The expression of EMT-associated markers and NOTCH4 protein in HGC-27 cells were detected by Western Blot; **(F)** The level of NOTCH4 mRNA in HGC-27 cells with different treatment were detected by RT-qPCR assay; **(G)** RNA FISH assay was performed to detect colocalization between lncRNA CADM2-AS1 and miR-5047, scale = 20 μm; Red, Cy3 fluorescent probe represented lncRNA CADM2-AS1; Green, FAM fluorescent probe represented miR-5047; Blue, DAPI represented the nucleus; Merge, a merge channel graph; **(H)** The predicted targeting sequence of miR-5047 on the lncRNA CADM2-AS1 and NOTCH4 mRNA. **(I)** Dual-luciferase reporter assay was performed to detect the targeting binding between lncRNA CADM2-AS1 and miR-5047; **(J)** Dual-luciferase reporter assay was performed to detect the targeting binding between NOTCH4 mRNA and miR-5047; Gray, represented the NC; Purple, represented 293T cells with miR-5047 overexpression; n = 3, *p < 0.05, **p < 0.01, ***p < 0.001, ****p < 0.0001.

Based on the above results, we found miR-5047 may be the mediator of lncRNA CADM2-AS1 upregulation of NOTCH4 in promoting GC metastasis. To verify this inference, we recovered the level of miR-5047 in HGC-27 pCADM2-AS1 cells by transfecting miR-5047 mimic (Figure 6A). The results of Transwell and Wound healing assay shown that, with miR-5047 recovered, the enhanced migration ability of HGC-27 by lncRNA CADM2-AS1 overexpression was also re-diminished (Figures 6B–D). Similarly, with miR-5047 mimic transfecting, the downregulated expression of epithelial cell marker (E-cadherin) by lncRNA CADM2-AS1 overexpression was increased, while the upregulated expression of mesenchymal cell markers (N-cadherin, Vimentin, Snail) by lncRNA CADM2-AS1 overexpression were decreased. Furthermore, the upregulated NOTCH4 was also re-decreased by miR-5047 mimic (Figure 6E). Meanwhile, RT-qPCR showed that the low level of NOTCH4 mRNA was restored with miR-5047 re-expression in HGC-27 pCADM2-AS1 (Figure 6F). In conclusion, the results preliminarily confirmed miR-5047 may be the mediator of lncRNA CADM2-AS1 upregulation of NOTCH4 in promoting GC metastasis, which still need more experimental verification.

To confirm that miR-5047 may be the mediator between lncRNA CADM2-AS1 and NOTCH4 mRNA, the cellular sublocalization of lncRNA CADM2-AS1 and miR-5047 was detected by RNA FISH assay firstly. The miR-5047 and lncRNA CADM2-AS1 were substantially co-localized in the cells, which signified that they had the possibility to combine with each other (Figure 6G). Meanwhile, bioinformatics prediction by RNA22 and Targetscan databases showed both lncRNA CADM2-AS1 and NOTCH4 mRNA might directly bind with the seed sequence (2-8 nucleotides) of miR-5047 (Figure 6H). Therefore, the Dual-luciferase reporter assay was performed to verify the binding between lncRNA CADM2-AS1 and miR-5047, as well as the binding between NOTCH4 mRNA and miR-5047. The result of Dual-luciferase reporter assay showed that overexpression of miR-5047 significantly reduced the fluorescence intensity of firefly luciferase from lncRNA CADM2-AS1 WT plasmid, while the fluorescence intensity of firefly luciferase from lncRNA CADM2-AS1 MUT plasmid had no obvious variation (Figure 6I). LncRNA CADM2-AS1 targeting bind with miR-5047. Identically, miR-5047 overexpression considerably reduced the fluorescence intensity of firefly luciferase from NOTCH4 WT plasmid, nevertheless, the fluorescence intensity of firefly luciferase from NOTCH4 MUT plasmid had no obvious change. The NOTCH4 was the target mRNA of miR-5047 (Figure 6J). To sum up, miR-5047 was the mediator of lncRNA CADM2-AS1 upregulation of NOTCH4 in promoting GC metastasis, which means that lncRNA CADM2-AS1 promoted GC metastasis by targeting the miR-5047/NOTCH4 signaling axis.

**FIGURE 7:**
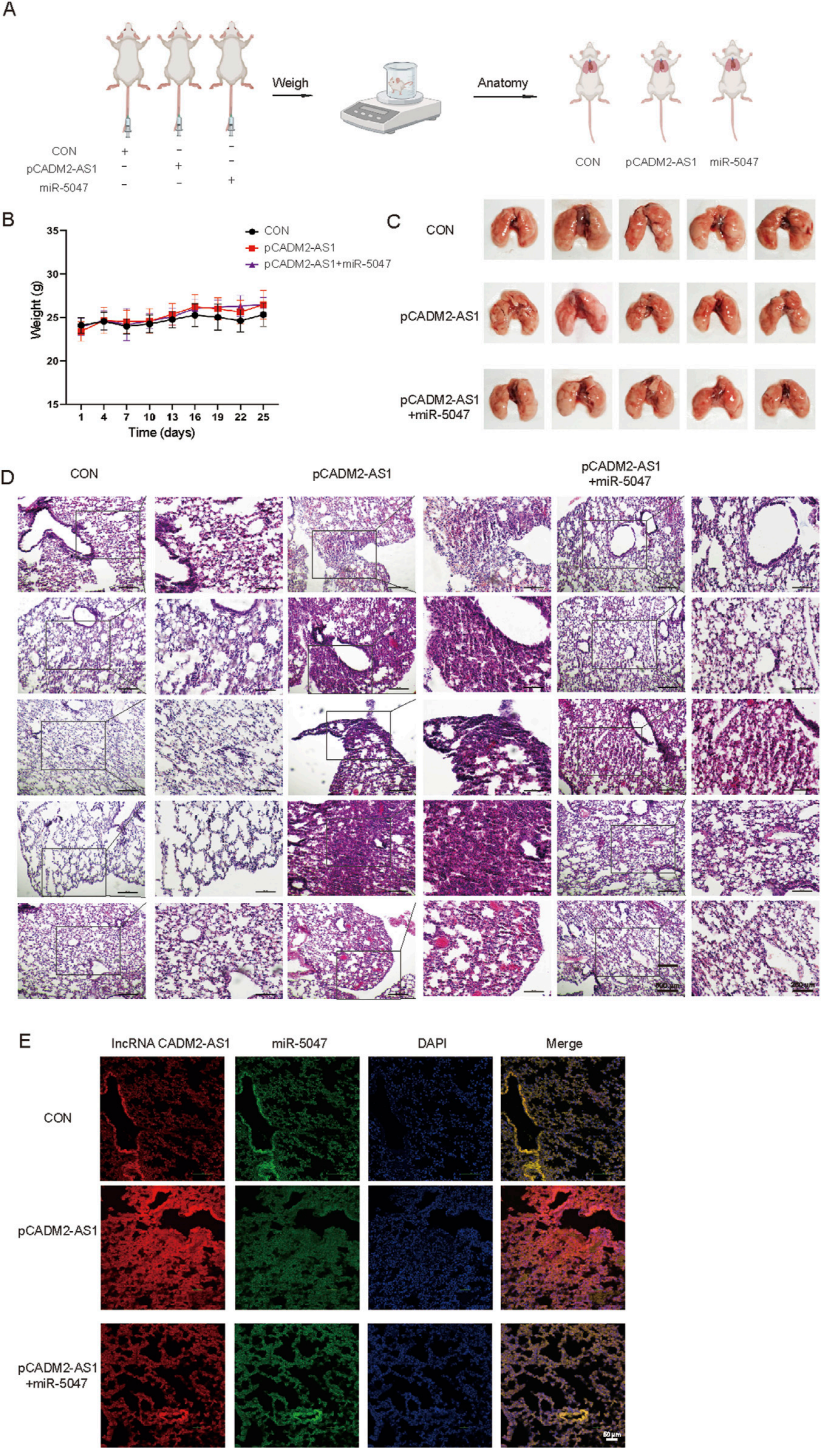
LncRNA CADM2-AS1 promoted the metastasis of GC cells via miR-5047/NOTCH4 signaling axis *in vivo*. **(A)** Mouse lung metastasis model construction pattern diagram; **(B)** With the lung metastasis model constructed, the mice were weighted in three groups once a week; **(C)** Brightfield map of fresh lung in pCADM2-AS1+miR-5047 group, pCADM2-AS1 group and control group; **(D)** HE staining of lung section in pCADM2-AS1+miR-5047 group, pCADM2-AS1 group and control group, scale = 500 μm; The figure on the right-side was a local magnification view, scale = 250 μm; n = 6; **(E)** RNA FISH assay was performed to detect the expression of lncRNA CADM2-AS1 and miR-5047 in GC, scale = 50 μm; Red, the Cy3 fluorescent probe represented lncRNA CADM2-AS1; Green, the FAM fluorescent probe represented miR-5047; Blue, DAPI represented nucleus; Merge, a merge channel graph.

To explore whether lncRNA CADM2-AS1 promote metastasis by miR-5047/NOTCH4 signaling axis, the lung metastasis model in NOD/SCID mouse was established by injecting HGC-27 CON, pCADM2-AS1 and pCADM2-AS1+miR-5047 cells into tail vein separately (Figure 7A). The weights were performed once a week. There was no significant difference in weight among the three groups (Figure 7B), while the lung nodules were obviously formed only in the lungs of pCADM2-AS1 group (Figure 7C). Compared to pCADM2-AS1 group, the recovery of miR-5047 in pCADM2-AS1 HGC-27 cells alleviated the ability of lncRNA CADM2-AS1 to promote metastasis of GC cells (Figures 7D, E). These phenomena proved lncRNA CADM2-AS1 can promote the metastasis of GC cells to the lung via miR-5047/NOTCH4 signaling axis *in vivo.*

### 3.6 LncRNA CADM2-AS1/miR-5047/NOTCH4 mRNA signaling axis was explored in clinical samples

**FIGURE 8:**
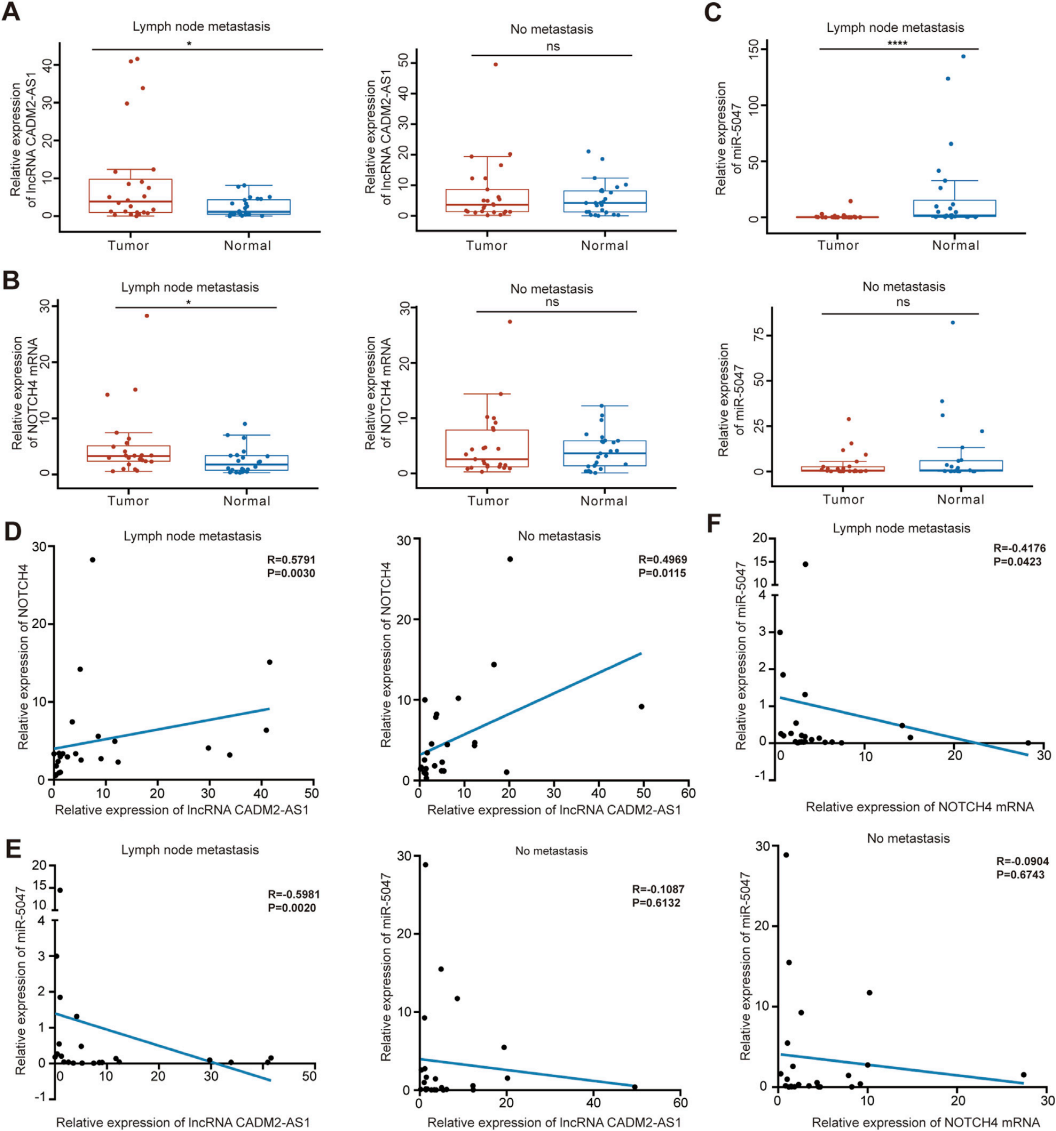
LncRNA CADM2-AS1/miR-5047/NOTCH4 mRNA signaling axis was explored in clinical tissues. **(A)**. The level of lncRNA CADM2-AS1 in GC tissues and tumor-adjacent were detected by RT-qPCR, the data were analyzed in GC tissues with or without lymph node metastasis separately and plotted in Hiplot Pro website; **(B)**. The level of NOTCH4 mRNA in GC tissues and tumor-adjacent were detected and analyzed; **(C)** The level of miR-5047 in GC tissues and tumor-adjacent were detected and analyzed; **(D)** The relationship between lncRNA CADM2-AS1 and NOTCH4 mRNA was analyzed by spearman analysis in GC tissues with or without lymph node metastasis respectively; **(E)** The relationship between lncRNA CADM2-AS1 and miR-5047 was analyzed by spearman analysis in GC tissues with or without lymph node metastasis respectively; **(F)** The relationship between NOTCH4 mRNA and miR-5047 was analyzed by spearman analysis in GC tissues with or without lymph node metastasis respectively; ns, not significantly different, *p < 0.05, ****p < 0.0001.

To verify the molecular mechanism of lncRNA CADM2-AS1 promoting GC metastasis by lncRNA CADM2-AS1/miR-5047/NOTCH4 mRNA signaling axis in metastatic GC patient tissues, 50 pair GC tissues and tumor-adjacent tissue were collected (Supplementary Table 1). There were 24 GC patients with lymph node metastasis and 25 GC patients without lymph node metastasis in the 50 pair tissues. Total RNA and miRNA were extracted respectively to detect the levels of lncRNA CADM2-AS1, NOTCH4 and miR-5047. It was found that the levels of lncRNA CADM2-AS1 and NOTCH4 mRNA in GC tissues with lymph node metastasis were notably higher than that in tumor-adjacent tissues respectively (Figures 8A, B), while the level of miR-5047 in GC tissues with lymph node metastasis was lower than that in tumor-adjacent tissues (Figure 8C). However, in tissues without lymph node metastasis, the expression of lncRNA CADM2-AS1, NOTCH4 mRNA and miR-5047 were no differences between GC tissues and tumor-adjacent tissues (Figures 8A–C). Moreover, it was discovered that the expression of lncRNA CADM2-AS1 was positively correlated with the expression of NOTCH4 mRNA (Figure 8D), while the expression of miR-5047 was negatively correlated with the expression of lncRNA CADM2-AS1 and NOTCH4 mRNA in GC tissues with lymph node metastasis (Figures 8E, F). However, in the tissues without lymph node metastasis, although the expression of lncRNA CADM2-AS1 was positively correlated with the expression of NOTCH4 mRNA, the relationships among miR-5047, lncRNA CADM2-AS1 and NOTCH4 mRNA has no statistical significance (Figures 8D–F). The regulation of lncRNA CADM2-AS1 on miR-5047 and NOTCH4 also existed in clinical tissues of GC with lymph node metastasis, which provided a new potential idea for the treatment of metastatic GC.

## 4 Conclusions

The number of new cases and deaths of GC were plentiful for long time (Siegel et al., 2022; Liu et al., 2022). It is becoming increasingly clear that lncRNAs are ubiquitous and novel classes of genes involved in GC (Okada et al., 2019). However, it has not been extensively studied how lncRNAs drive metastasis of GC. After profiling lncRNAs dysregulated between GC lymph node metastasis and normal tissues via lncRNA-seq, we identified plenty of lncRNAs with continuously elevated or decreased expression patterns in these tissues. Among them, the expression of lncRNA CADM2-AS1 increased obviously, which suggested that lncRNA CADM2-AS1 may involve in metastasis of GC.

The further study demonstrated that lncRNA CADM2-AS1 knockdown inhibited migration abilities of GC cells, while lncRNA CADM2-AS1 overexpression promoted the metastasis of GC cells. Moreover, epithelial cell marker (E-cadherin) was upregulated and mesenchymal cell markers (N-cadherin, Vimentin, Snail) were downregulated with lncRNA CADM2-AS1 knockdown; lncRNA CADM2-AS1 overexpression also enhanced the expression of N-cadherin, Vimentin and Snail and decreased the expression of E-cadherin, which were necessary for GC cell metastasis. In addition, the results of lung metastasis models shown that overexpression of lncRNA CADM2-AS1 increased the number of pulmonary nodules. All these data proved that high expression of lncRNA CADM2-AS1 facilitate GC cells metastasis *in vitro* and *in vivo*.

However, there have been not enough reports on the role of lncRNA CADM2-AS1 in GC metastasis. The location of lncRNA CADM2-AS1 in cytoplasm suggested that it might act through the miRNA/mRNA signaling axis. In addition, the result of RNA-seq revealed that overexpression of lncRNA CADM2-AS1 could markedly increase NOTCH4 mRNA expression. Therefore, we speculated that lncRNA CADM2-AS1 improved metastasis of GC by raising NOTCH4 mRNA.

As expected, our results confirmed our hypothesis. NOTCH4 was a significant promoter of GC metastasis, and lncRNA CADM2-AS1 overexpression could upregulate NOTCH4 mRNA by down-regulating miR-5047. Meanwhile, the results of Dual-luciferase reporter assays showed that miR-5047 bound to both lncRNA CADM2-AS1 and NOTCH4. Besides, with miR-5047 recovered in HGC-27 pCADM2-AS1, the enhanced migration ability of HGC-27 by lncRNA CADM2-AS1 upregulated NOTCH4 mRNA was also re-diminished *in vitro* and *in vivo*. MiR-5047 was the mediator of lncRNA CADM2-AS1 upregulation of NOTCH4 in promoting GC metastasis.

What is more, lncRNA CADM2-AS1 expression was positively correlated with the expression of NOTCH4 mRNA in GC tissues with lymph node metastasis, while miR-5047 expression was negatively correlated with the expression of lncRNA CADM2-AS1 and NOTCH4 mRNA. The regulation of lncRNA CADM2-AS1 on miR-5047 and NOTCH4 also existed in clinical tissues of GC with lymph node metastasis, which give lncRNA CADM2-AS1 the potential to a prognostic and predictive biomarker in treatment of metastatic GC.

**FIGURE 9:**
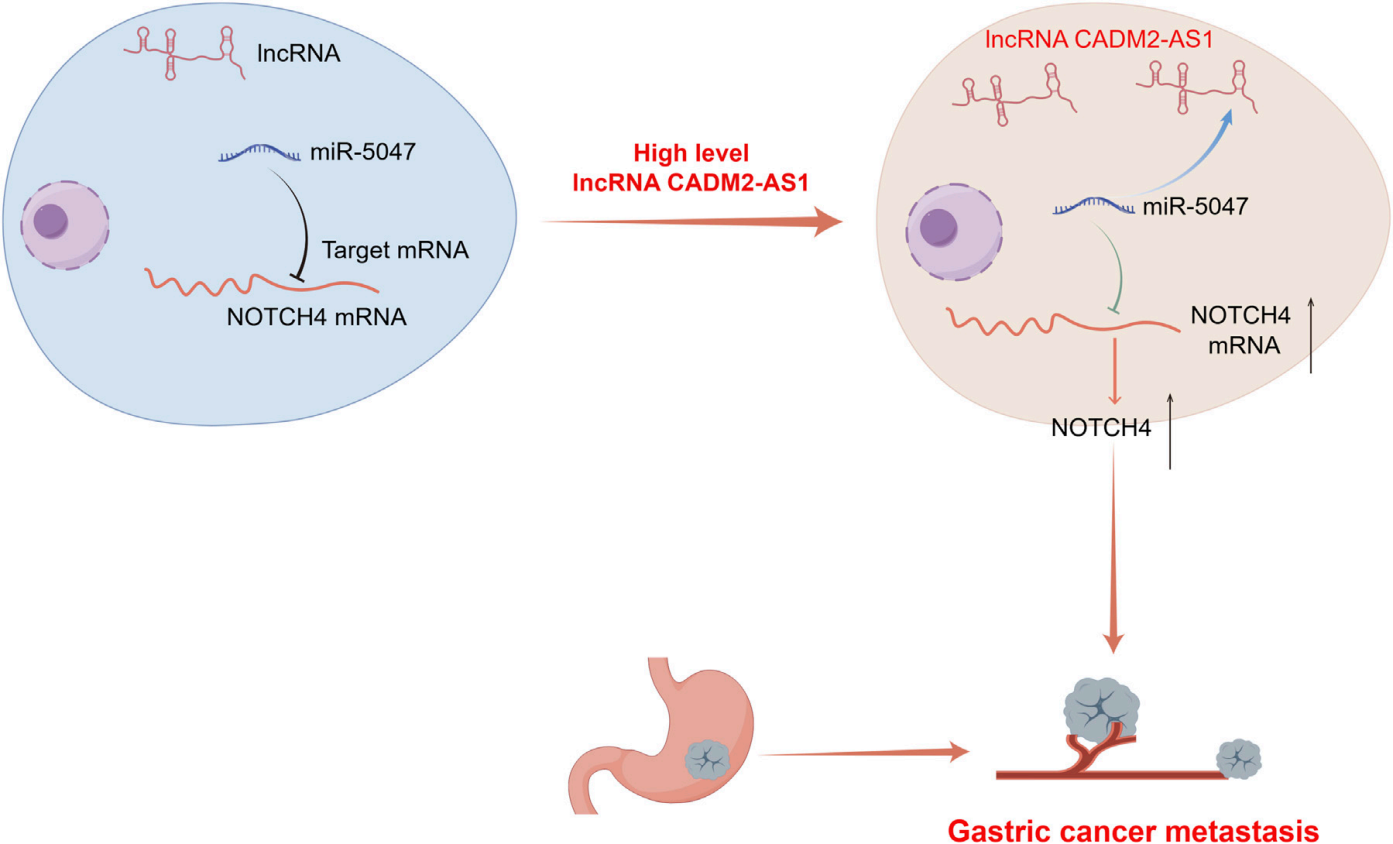
A diagram for this article. Elevated expression of LncRNA CADM2-AS1 promoted metastasis in GC by targeting the miR-5047/NOTCH4 signaling axis. Diagram drew by Figdraw.

In conclusion, high lncRNA CADM2-AS1 expression upregulated NOTCH4 mRNA to promote metastasis of GC cells by silencing miR-5047 both *in vitro* and *in vivo* (Figure 9). The relationship among lncRNA CADM2-AS1, miR-5047 and NOTCH4 was further detected and verified in metastatic GC patient tissues. These results offered strong support for the hypothesis that LncRNA CADM2-AS1 may be a potential target for metastasic GC prognosis in the clinic. Targeting reduced LncRNA CADM2-AS1 may contribute to inhibit the progression of GC metastasis, which has great significance to propose a new treatment plan for metastasic GC.

## 5 Discussion

In the present study, the overexpression of lncRNA CADM2-AS1 was found and verified from clinic GC patient tissues, which endows this study with high research significance because it starts from clinical phenomena. Based on the exploration the mechanism that high expression of lncRNA CADM2-AS1 promote metastasis of GC cells by upregulated the target mRNA of miR-5047, NOTCH4, *in vitro* and *in vivo*. The relationship among lncRNA CADM2-AS1, miR-5047 and NOTCH4 was further detected and verified in metastatic GC patient tissues. These results offered strong support for the hypothesis that lncRNA CADM2-AS1 may be a potential target for metastasic GC prognosis in the clinic. Therefore, inhibition of GC metastasis via precisely controlling lncRNA CADM2-AS1 levels might represent as potential therapeutic methods. Uncovering the precise role of lncRNA CADM2-AS1/miR-5047/NOTCH4 regulatory axis in GC metastasis will not only increase our knowledge of noncoding RNAs-regulated therapeutic effect in cancer metastasis and the underlying regulatory mechanism, but also help develop more efficient strategies to reduce the incidence of GC metastasis.

## Data Availability

The datasets presented in this study can be found in online repositories. The mRNA-seq data have been submitted in SRA database, and the number was PRJNA1115977.

## Abbreviations

ASO, The antisense oligonucleotide; ceRNAs, Competing endogenous RNAs; GC, Gastric cancer; HE staining, Hematoxylin-eosin staining; lncRNAs, Long noncoding RNAs; miRNAs, MicroRNAs; NOTCH4, neurogenic locus notch homolog protein; 4RPMI, Roswell Park Memorial Institute; RT-qPCR, Quantitative reverse transcription PCR.
