# Supplementary material for: The lncRNA CADM2-AS1 promotes gastric cancer metastasis by binding with miR-5047 and activating NOTCH4 translation
Source: Front Pharmacol. 2024 Sep 6;15:1439497. doi: 10.3389/fphar.2024.1439497 (PMC11412803; doi:10.3389/fphar.2024.1439497)
Supplement: Supplementary file 1 [file Table1.DOCX]

# Supplementary Table 1

Supplementary Table 1 Information of GC patients

| Factor | The patient proportion (%) |
| --- | --- |
| Gender | |
| Man | 32 (64%) |
| Female | 10 (20%) |
| Unknown | 8 (16%) |
| Age | |
| <60 | 17 (34%) |
| ≥60 | 24 (48%) |
| Unknown | 9 (18%) |
| Lymph node metastasis | |
| Yes | 24 (48%) |
| No | 25 (50%) |
| Unknown | 1 (2%) |
| Differentiation degree | |
| Low | 24 (48%) |
| Low-medium | 7 (14%) |
| Medium | 16 (32%) |
| High | 1 (2%) |
| Unknown | 2 (4%) |
