# Supplementary figures and images for: The lncRNA CADM2-AS1 promotes gastric cancer metastasis by binding with miR-5047 and activating NOTCH4 translation
Source: Front Pharmacol. 2024 Sep 6;15:1439497. doi: 10.3389/fphar.2024.1439497 (PMC11412803; doi:10.3389/fphar.2024.1439497)

Figure 2

E

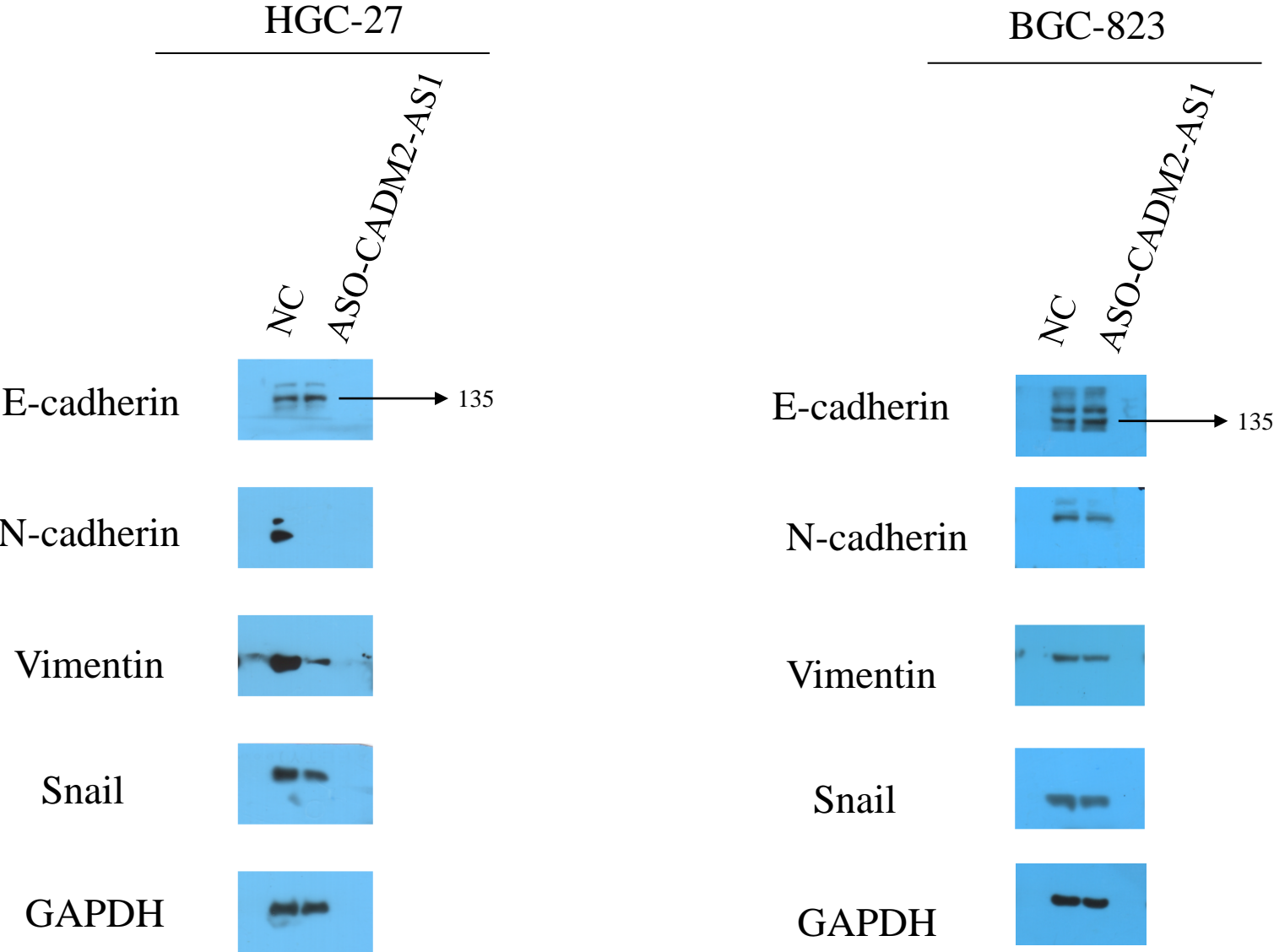

Figure 3

E

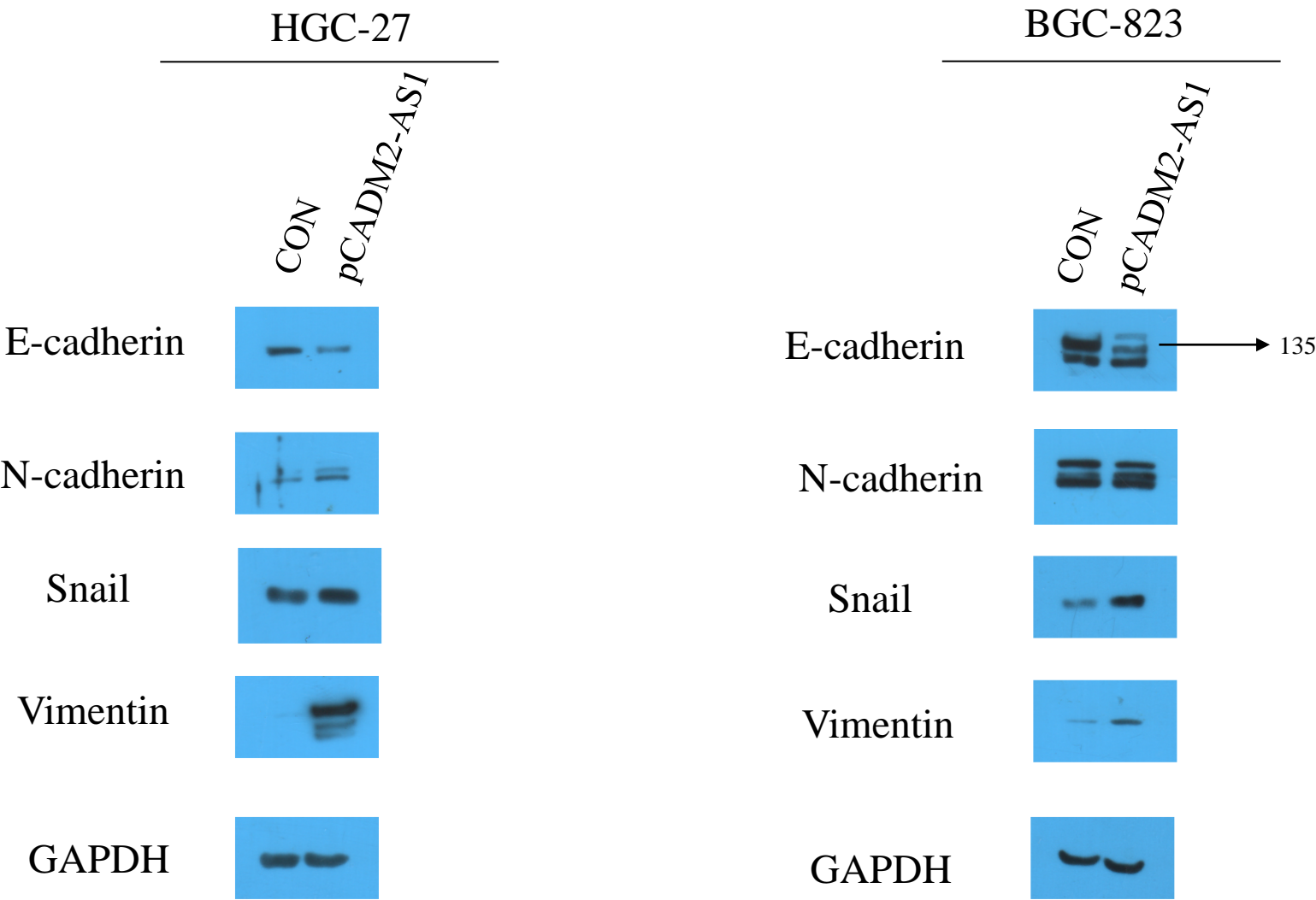

Figure 5

G

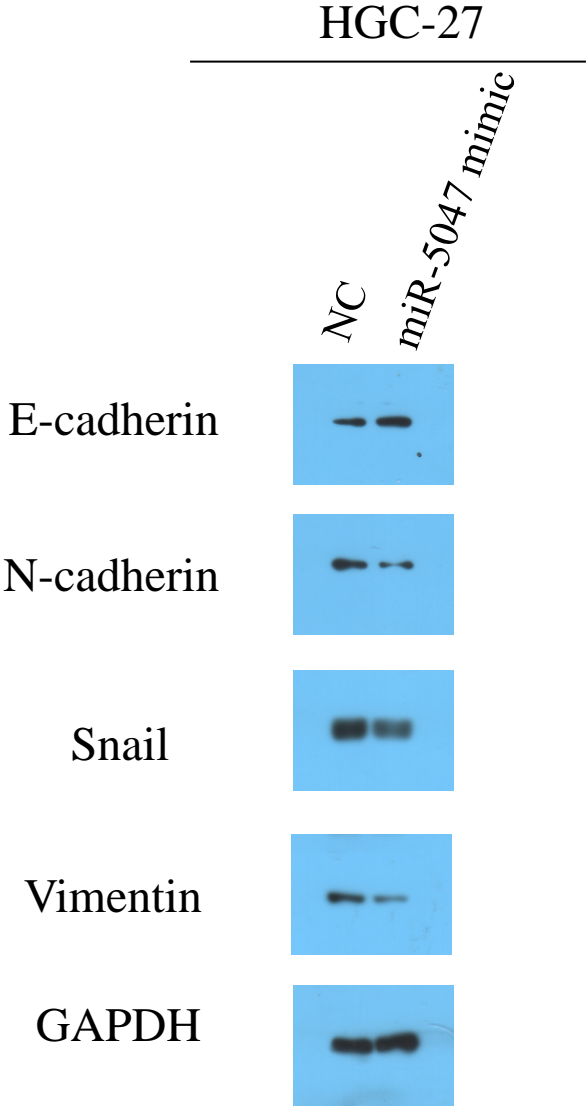

Figure 6

E

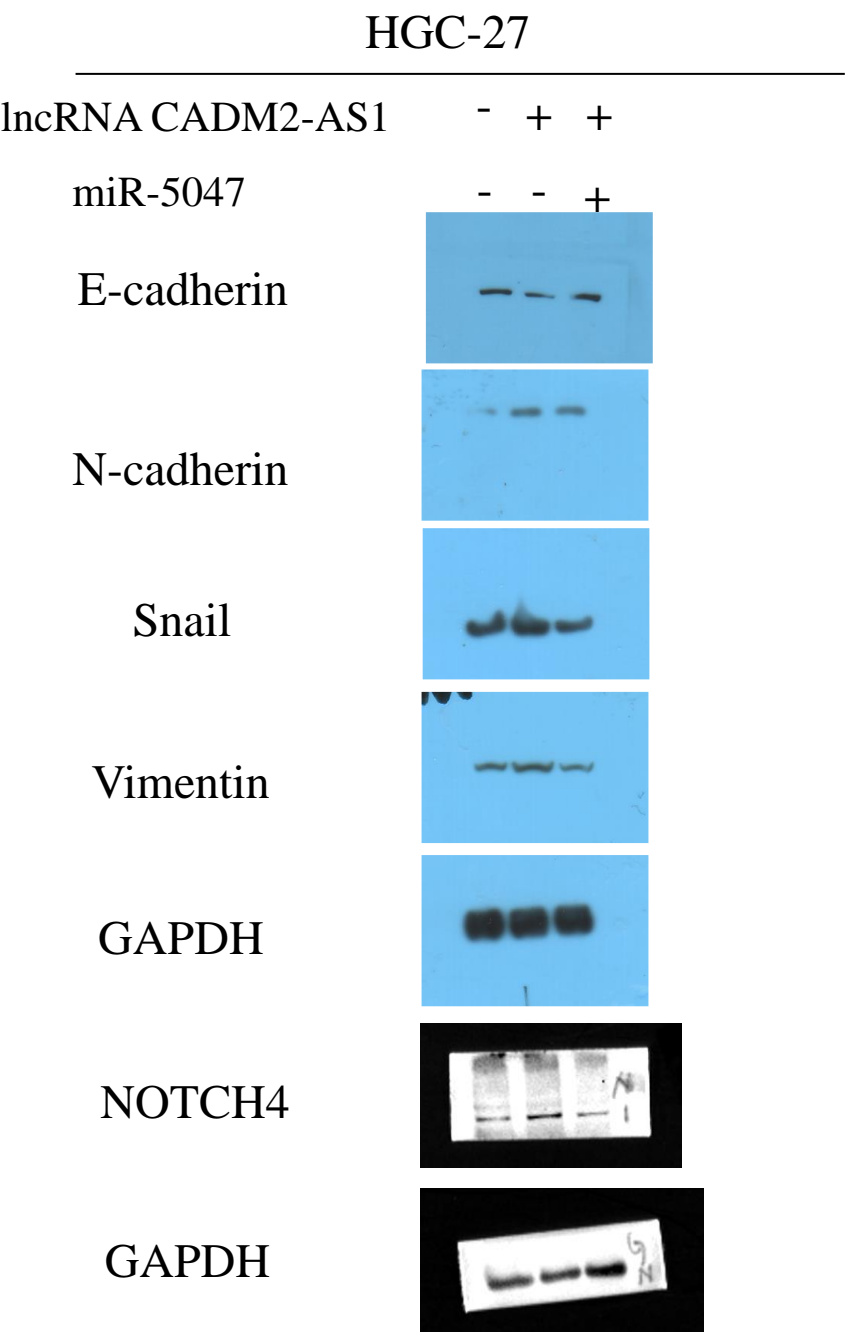

Supplement: Supplementary file 2 [file DataSheet1.PDF]
